# Towards sustainable electrochemistry: green synthesis and sintering aid modulations in the development of BaZr_0.87_Y_0.1_M_0.03_O_3−δ_ (M = Mn, Co, and Fe) IT-SOFC electrolytes

**DOI:** 10.3389/fchem.2023.1322475

**Published:** 2023-11-27

**Authors:** Qurat ul Ain, Muneeb Irshad, Muhammad Salim Butt, Asif Nadeem Tabish, Muhammad Bilal Hanif, Muhammad Ali Khalid, Rabia Ghaffar, Muhammad Rafique, Syeda Dur E. Shawar Kazmi, Khurram Siraj, Amal A. Abdel Hafez, Hisham S. M. Abd-Rabboh, Zuzana Zmrhalova, Elena A. Filonova, Dmitry A. Medvedev, Martin Motola

**Affiliations:** ^1^ Department of Physics, University of Engineering and Technology, Lahore, Pakistan; ^2^ Department of Electrical Engineering, University of Engineering and Technology, New Campus, Lahore, Pakistan; ^3^ Department of Chemical Engineering, University of Engineering and Technology, New Campus, Lahore, Pakistan; ^4^ Department of Inorganic Chemistry, Faculty of Natural Sciences, Comenius University Bratislava, Bratislava, Slovakia; ^5^ State Key Laboratory for Mechanical Behavior of Materials, School of Materials Science and Engineering, Xi’an Jiaotong University, Xi’an, Shaanxi, China; ^6^ State Key Laboratory of Multiphase Flow in Power Engineering, School of Energy and Power Engineering, Xi’an Jiaotong University, Xi’an, China; ^7^ Division of Science and Technology, Department of Botany, University of Education, Lahore, Pakistan; ^8^ Department of Physics, University of Sahiwal, Sahiwal, Pakistan; ^9^ Department of Chemistry, Faculty of Science, King Khalid University, Abha, Saudi Arabia; ^10^ Center of Materials and Nanotechnologies, Faculty of Chemical Technology, University of Pardubice, Pardubice, Czechia; ^11^ Institute of Natural Sciences and Mathematics, Ural Federal University, Ekaterinburg, Russia; ^12^ Hydrogen Energy Laboratory, Ural Federal University, Ekaterinburg, Russia; ^13^ Laboratory of Electrochemical Devices Based on Solid Oxide Proton Electrolytes, Institute of High Temperature Electrochemistry, Ekaterinburg, Russia

**Keywords:** perovskite, green synthesis, SOFC, proton conductor, barium zirconate, electrochemical performance

## Abstract

In this study, BaZr_0.87_Y_0.1_M_0.03_O_3−δ_ perovskite electrolytes with sintering aids (M = Mn, Co, and Fe) were synthesized by a sustainable approach using spinach powder as a chelating agent and then compared with chemically synthesized BaZr_0.87_Y_0.1_M_0.03_O_3−δ_ (M = Mn, Co, and Fe) electrolytes for intermediate temperature SOFCs. This is the first example of such a sustainable synthesis of perovskite materials with sintering aids. Structural analysis revealed the presence of a cubic perovskite structure in BaZr_0.87_Y_0.1_M_0.03_O_3−δ_ (M = Mn, Co, and Fe) samples synthesized by both green and conventional chemical methods. No significant secondary phases were observed in the samples synthesized by a sustainable approach. The observed phenomena of plane shift were because of the disparities between ionic radii of the dopants, impurities, and host materials. The surface morphology analysis revealed a denser microstructure for the electrolytes synthesized via green routes due to metallic impurities in the organic chelating agent. The absence of significant impurities was also observed by compositional analysis, while functional groups were identified through Fourier-transform infrared spectroscopy. Conductivity measurements showed that BaZr_0.87_Y_0.1_M_0.03_O_3−δ_ (M = Mn, Co, and Fe) electrolytes synthesized by oxalic acid have higher conductivities compared to BaZr_0.87_Y_0.1_M_0.03_O_3−δ_ (M = Mn, Co, and Fe) electrolytes synthesized by the green approach. The button cells employing BaZr_0.87_Y_0.1_Co_0.03_O_3−δ_ electrolytes synthesized by the chemical and green routes achieved peak power densities 344 and 271 mW·cm^−2^ respectively, suggesting that the novel green route can be applied to synthesize SOFC perovskite materials with minimal environmental impact and without significantly compromising cell performance.

## 1 Introduction

Energy is a crucial element in the long-term development and wellbeing of all nations. As the world’s population has grown and technological industrialization has progressed, energy has become an indispensable requirement for daily life. Energy consumption is also essential for economic development and prosperity, especially for electricity generation and industrial use (Babar et al., 2022; Hanif et al., 2023a; Antonova, 2023; Gordeev and Porotnikova, 2023; Shaheen et al., 2023). Fossil fuels are still the primary sources of energy but concerns about greenhouse gas emissions and climate change have increased, forcing the world to explore renewable and sustainable energy sources (Hanif et al., 2022; Irshad et al., 2022; Demin and Bronin, 2023; Pikalova et al., 2023). Fuel cells, particularly solid oxide fuel cells (SOFCs), have become prominent contenders amongst alternative energy sources because of their efficiency, ability to use multiple fuels, and little or no greenhouse gas emissions (Rafique et al., 2022; Hanif et al., 2023b; Khan et al., 2023; Rauf et al., 2023; Tarasova et al., 2023; Babar et al., 2024). In the last decade, special attention has been focused on proton-conducting SOFCs owing to their high protonic conductivity compared to oxygen conduction because of the small size of protons with their low activation energy (Irshad et al., 2023; Mehran et al., 2023).

The electrolyte is an essential component of SOFCs, and its improvements are critical in reducing operating temperatures and achieving good chemical stability and ionic conductivity at the same time. Many ionic conductors, including YSZ, doped gallates and doped CeO_2_, etc., are investigated as SOFC electrolytes. Nevertheless, these electrolytes need a substantial amount of activation energy and elevated temperature in order to achieve good conductivity. Proton conductors have the potential to be suitable for use as electrolytes due to their favorable proton conductivity at low temperatures (Irshad et al., 2016; Mosiałek et al., 2023). Perovskite materials (ABO_3_) have attracted considerable attention as electrolyte materials because they can surpass the constraints of traditional electrolytes. The highly conducting perovskite electrolytes can transport ions more efficiently across the material. The aforementioned attribute can enhance the SOFC performance device by facilitating swift ions transport and minimizing resistances. In addition, these electrolytes are also more stable than conventional electrolytes in reductive/oxidative atmospheres at high operating temperatures (Irshad et al., 2023).

Doped barium zirconate (BaZrO_3_) and barium cerate (BaCeO_3_) are commonly used proton conducting electrolytes based on the perovskite structure (ABO_3_). Doped BaCeO_3_exhibits high conductivity, however it lacks chemical stability in CO_2_ and humid atmospheres, whereas doped BaZrO_3_ exhibits high stability in these atmospheres, but have lower ionic conductivity (Goulart et al., 2021; Rasaki et al., 2021; Zhang and Hu, 2021). Various sintering aids, synthesis routes and doping approaches are used to increase the density and conductivity of barium zirconate-based materials (Jiao et al., 2019). Introducing rare earth elements to the B-site creates oxygen vacancies, which in turn enhances ionic conductivity. Consequently, perovskites doped with rare earth (RE) elements have garnered interest because of their elevated levels of ionic, proton, or mixed conductivities (Irshad et al., 2021; Rasaki et al., 2021). The addition of dopants in perovskites and especially in BaZrO_3_ enhances the proton mobility and therefore boosts proton conductivity at intermediate temperatures (Nayak and Sasmal, 2023). The meticulous choice of dopants along with fabrication techniques could ascertain the effective formation of conductive and stable BaZrO_3_-based electrolytes (Hossain et al., 2021a). RE-doped BaZrO_3_ exhibit significant protonic conductivity at low temperatures making them a suitable choice. However, there are still existing problems, such as the limited ability of protons to be absorbed and the insufficient number of catalytic sites which needs to be addressed. Furthermore, achieving dense BaZrO_3_-based electrolytes necessitates elevated sintering temperature and prolonged sintering duration. High sintering temperatures results in evaporation of BaO, causing a decrease in grain and grain boundary conductivity due to ternary phase formation (Ueno et al., 2019). Also, BaZrO_3_ has a grain boundary blocking property caused by a space charge effect which segregates the charged defects near structurally distorted region of the grain boundary (Uthayakumar et al., 2020). Despite substantial research on the proton conduction of doped BaZrO_3_, the exact processes underlying the conduction of doped BaZrO_3_ is remain poorly understood (Vera et al., 2021). It is however an established fact that synthesis approaches, selection of dopants and sintering aids are crucial role for promoting the desired properties of RE-BaZrO_3_ materials (Hossain et al., 2021b).

Researchers have reported the use of sintering aids to decrease the sintering temperature while upholding the attributes of the SOFC materials. Soares et al. (2021) investigated ZnO as a sintering aid with different stoichiometric ratios in Ba(Zr,Y)O_3−δ_ to improve the densification, bulk proton conductivity, and high hydration enthalpy. Ho-Il Ji et al. investigated 1 and 4 mol% of CuO and ZnO as sintering aids to reduce the sintering temperature from 1,700°C to 1,500°C and 1,300°C, respectively and achieved relative densities above 97% (Ji et al., 2021). The use of transition metals (Sc, Zn, Co, Cu, and Fe, etc.) as co-dopants can improve the sinterability and thus increase the densification (Xie et al., 2018; Ueno et al., 2019; Aarthi and Babu, 2020). Xie et al. (2018) observed the co-doing effect of Gd-Zn on barium zirconate sintered at 1,300°C–1,500°C, which improved the mechanical performance, sinterability, hardness, and conductivity, achieving a conductivity of 2.54 × 10^−3^ S ·cm^−1^ and power density of 282 mW·cm^−2^.

Diverse synthetic pathways, encompassing both physical and chemical methodologies, have been utilized to synthesize of SOFC materials. However, these procedures have a substantial ecological impact (Zheng et al., 2009; Zhang and Hu, 2021; Tong et al., 2022). Little or no effort has been made to synthesize perovskite SOFC materials by green synthesis due to the presence of organic and inorganic impurities that significantly hinder or degrade the performance of SOFCs. Spinach was used as a chelating agent to leverage the synergistic role of both its biomolecules and natural oxalic acid (present in high content) as a reducing and capping agent. Furthermore, the metallic impurities it contains might act as a sintering aid, resulting in the better densification at a lower sintering temperature. Spinach is frequently associated with a high oxalic acid concentration, with a quantity of around 3.45 ± 0.22 mg·g^−1^ (Uwah et al., 2011). The metal content in spinach is Zn, 6.10 ± 0.12; Mn, 10.50 ± 0.90; As, 0.90 ± 0.26; Pb, 2.40 ± 0.16; Cu, 0.88 ± 0.07 and Cd, 0.26 ± 0.02 μg·g^−1^ (Osinkin, 2023). Furthermore, there is no literature to date where perovskite materials with sintering aids have been developed by green synthesis. In the current project, a novel sustainable approach will be developed to synthesize the perovskite materials with sintering aids using biomolecules and natural chelating agents as reducing and capping agents with minimal impurities. BaZr_0.87_Y_0.1_M_0.03_O_3−δ_ perovskite electrolytes co-doped with sintering aids (M = Co, Fe, Mn) have been successfully synthesized via green and chemical routes using bio- and chemical chelating agents respectively. The BaZr_0.87_Y_0.1_M_0.03_O_3−δ_ (M = Co, Fe, Mn) samples synthesized with oxalic acid is denoted as BZYCo (OA), BZYFe (OA), and BZYMn (OA), respectively. Similarly, the BaZr_0.87_Y_0.1_M_0.03_O_3−δ_ (M = Co, Fe, Mn) samples synthesized with spinach is labeled as BZYCo (SP), BZYFe (SP), and BZYMn (SP), respectively.

## 2 Experimentation

**FIGURE 1:**
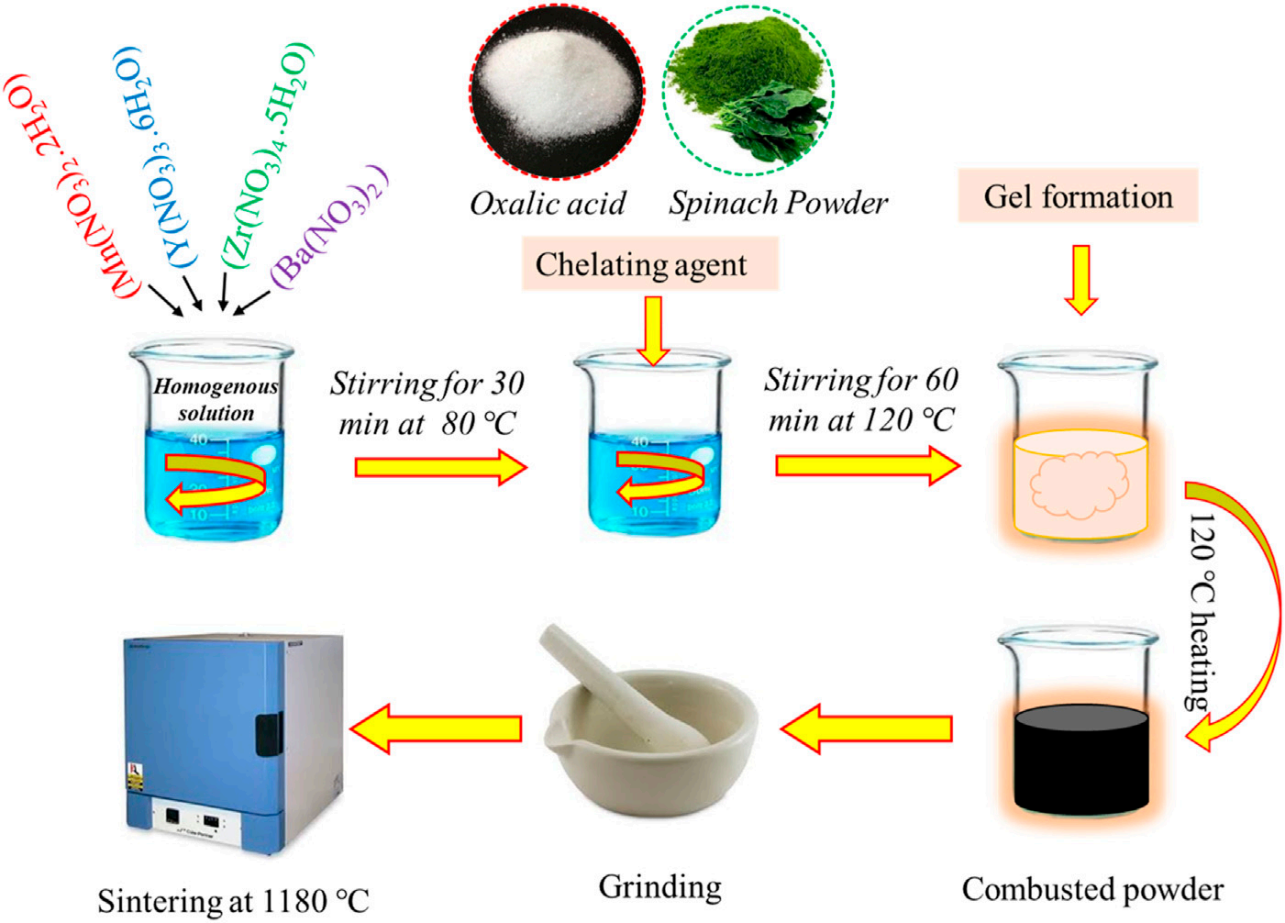
Schematic representation for the synthesis of BaZr_0.87_Y_0.1_M_0.03_O_3−δ_ (BZYM) electrolyte.

BaZr_0.87_Y_0.1_M_0.03_O_3−δ_ (BZYM) with transition metal as B-site co-dopants (M = Co, Fe, Mn) were synthesized by auto-combustion method using different chelating agents, i.e., oxalic acid and spinach dried leaves powder. The stoichiometric amount of [Ba(NO_3_)_2_], [Zr(NO_3_)_4_⋅5H_2_O], [Y(NO_3_)_3_⋅6H_2_O], [Mn(NO_3_)_2_⋅2H_2_O], [Fe(NO_3_)_3_⋅9H_2_O] and [Co(NO_3_)_2_⋅6H_2_O] were dissolved in distilled water on a hot plate with a magnetic stirrer at 80°C for 30 min and then stirred at 120°C for 60 min to obtain a homogeneous solution. 20 wt.% of oxalic acid was dissolved in the homogeneous solution as a chelating agent. The solution was stirred continuously at 90°C to form a gel which was auto combusted to powder form. The powder obtained was sintered at 1,180°C for 6 h. The same stoichiometric amount of nitrate was used to prepare another homogeneous solution in the same way, with another chelating agent, i.e., 20 wt.% of spinach dry leaves powder. Again, the homogeneous solution was stirred at 80°C for 30 min and then stirred at 120°C for 60 min to produce a gel that auto combusted into powder. These powder samples were also sintered at 1,180°C for 6 h. The pellets were formed using a hydraulic press at 100 MPa for 5 min (Figure 1).

### 2.1 Material characterizations

The analysis of the BaZr_0.87_Y_0.1_M_0.03_O_3−δ_ (M = Co, Fe, Mn) was conducted through X-Ray diffractometer (XRD; Bruker D8, Netherlands), Thermogravimetric analysis (TGA; STA 449 F5 Jupiter, Netzsch, Selb, Germany), Scanning electron microscopy (FESEM JEOL, Japan), Energy dispersive analysis (EDX), Fourier Transform Infrared spectroscopy (FTIR; JASCO 4600). The ionic conductivity was obtained through four probe DC method at 200°C–600°C. Ionic conductivity was calculated using equation.

σ=LRA (1)

The Arrhenius equation was used to determine the relation between temperature and conductivity.

$$\sigma =\sigma_{{}^{\circ}}\exp\left(-E_{a}/KT\right)$$ (2)

### 2.2 Cell fabrication

The electrochemical performances of BZYM (M = Co, Fe, Mn) electrolytes, utilizing oxalic acid and spinach powder as chelating agents, were assessed. Pellets with a diameter of 13 mm and a thickness of 0.6 mm were prepared using a hydraulic press under a pressure of 200 MPa. BSCF and Ni-BZY were used as cathode and anode with BaZr_0.87_Y_0.1_M_0.03_O_3−δ_ (M = Co, Fe, Mn) as electrolyte for the electrochemical measurements of the button cell. Humidified (∼3% H_2_O) hydrogen is supplied as fuel at the anode at 50 mL·min^−1^ while oxygen is supplied as an oxidant at the cathode.

## 3 Results and discussion

### 3.1 Crystal structure analysis

**FIGURE 2:**
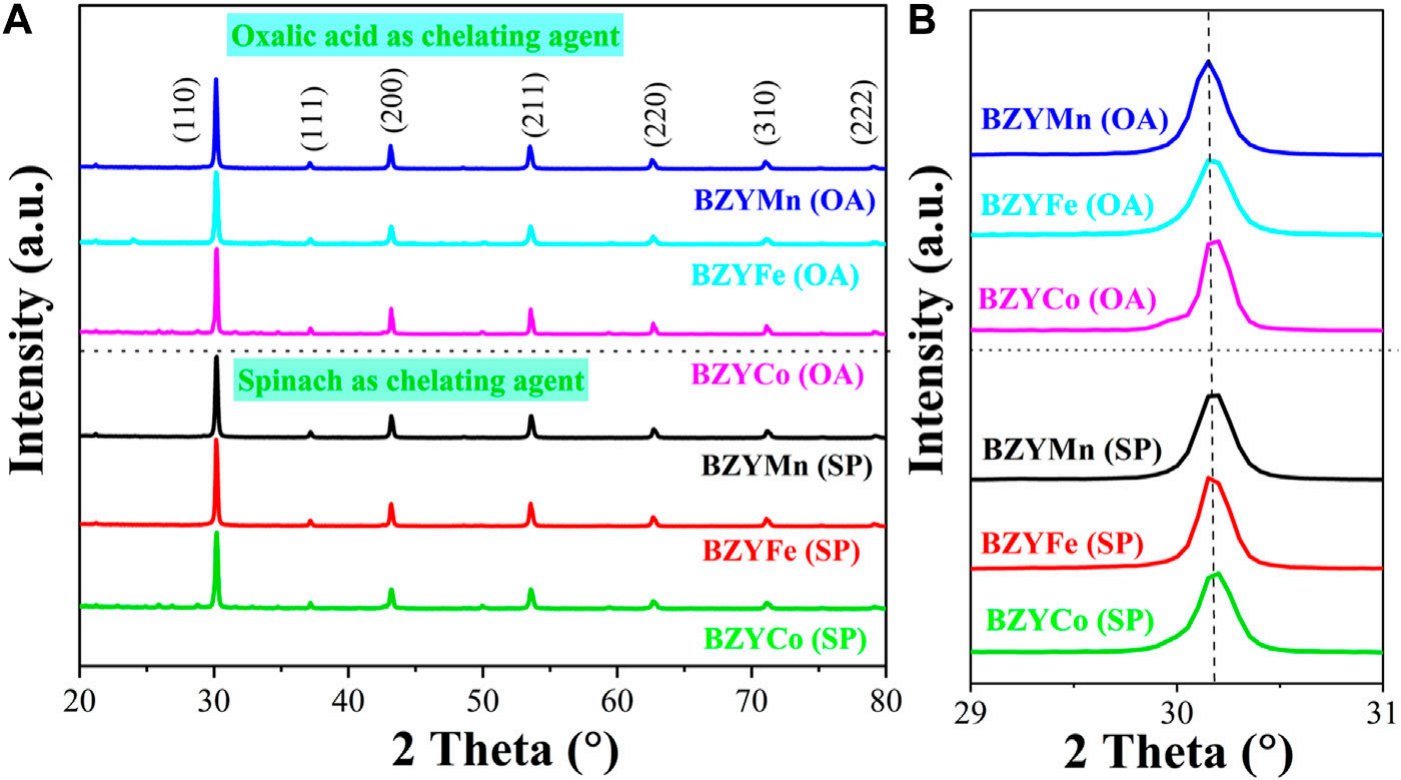
XRD spectra of BZYM (M = Co, Fe, Mn) electrolytes synthesized by chemical (oxalic acid) and green (spinach) routes **(A)** and enlarged (110) plane **(B)**.

Figures 2A, B shows the XRD spectra of BZYM (M = Co, Fe, Mn) perovskite electrolyte, synthesized through chemical and green routes. The presence of diffraction peaks at (110), (111), (200), (211), (220), and (310) provides confirmation that all BZYM electrolytes exhibit a cubic perovskite structure and belongs to the primitive space group *Pm3m* (ICDD# 98 010 7880) (Jiao et al., 2019). The absence of significant secondary phases of zirconium, yttrium, or transition metals confirms single cubic perovskite structure. The lack of subsequent phases also confirms the solubility of transition metals (Mn, Fe, Co) in the host lattice synthesized by both chemical and green synthesis methods.

The orientation of the plane (110) corresponds to the alteration in lattice parameter, resulting in either an increase (cell volume expansion) or reduction (cell volume contraction) depending on the ionic radii and dopant concentration (Irshad et al., 2021). BZY shows a peak shift to a lower angle compared to the pure barium zirconate, which has an intense diffraction peak at 2Ө = 30⁰, because of disparity between ionic radii of host (r = 0.72 Å for Zr^+4^_VI_) and dopant (for Y^+3^_VI_ r = 0.9 Å) (Ueno et al., 2019; Vera et al., 2021; Irshad et al., 2022). In our case, this peak shift occurs at 2Ө = 30.15⁰ for (110) plane of BZYCo (OA), BZYFe (OA), BZYMn (OA), BZYCo (SP), BZYFe (SP) and BZYMn (SP). This shift of the peak to a higher angle compared to BZY, indicating lattice contraction due to the incorporation of small ionic radii of secondary dopants, i.e., Co^+3^_VI(HS)_ (0.61 Å), Fe^+3^_VI(HS)_ (0.65 Å), and Mn^+3^_VI(HS)_ (0.66 Å) into BZY compared to the host element Zr (0.72 Å) and dopant Y (0.9 Å). The crystallite sizes of BZYCo (OA), BZYFe (OA), BZYMn (OA), BZYCo (SP), BZYFe (SP) and BZYMn (SP) were calculated using the Scherer formula as follows:

$$D=\frac{K\lambda }{\beta \cos Ө}$$ (3)

The crystallite sizes of the synthesized materials BZYM (M = Co, Fe, Mn) with chemical and green routes are given in Table 1.

**TABLE 1:** Average crystallite size of BZYM (M = Co, Fe, Mn) electrolytes synthesized with oxalic acid (OA) and spinach dry leaves powder (SP) as chelating agents.

Material	Dopant	Average crystallite size (nm)
BZYCo (OA)	Co	25
BZYFe (OA)	Fe	28
BZYMn (OA)	Mn	29
BZYCo (SP)	Co	23
BZYFe (SP)	Fe	26
BZYMn (SP)	Mn	30

### 3.2 Surface morphological analysis

**FIGURE 3:**
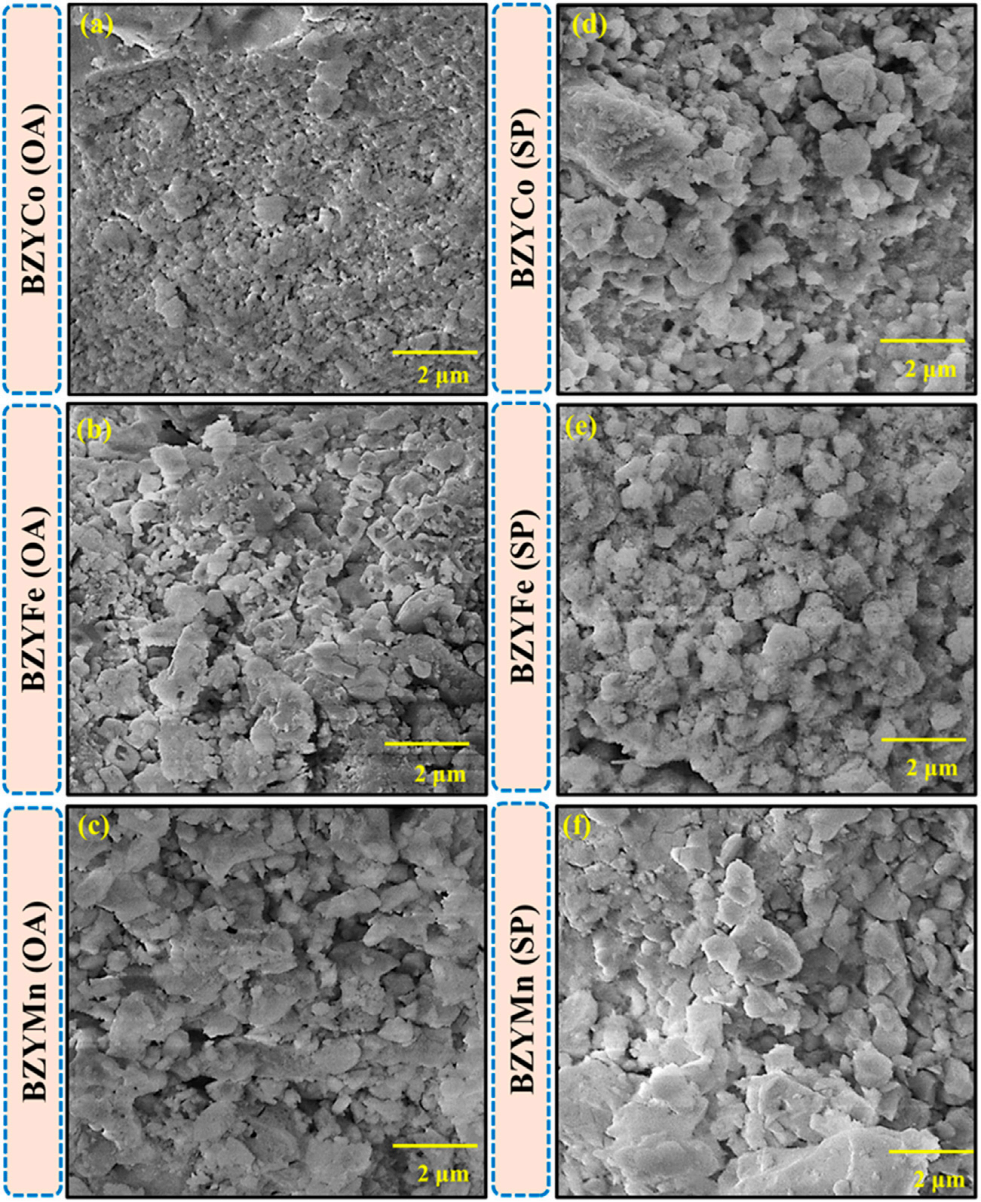
Surface morphology of BZYM (M = Co, Fe, Mn) electrolytes synthesized by green **(A–C)** and chemical routes **(D–F)**.

Figures 3A–F show the SEM micrographs of the synthesized BZYM (M = Co, Fe, Mn) using spinach and oxalic acid as chelating agents respectively. It is clear that all the synthesized materials have a dense microstructure. However, BZYM (M = Co, Fe) synthesized by the green route are slightly denser compared to BZYM (M = Co, Fe) synthesized by the chemical approach which is attributable to low concentration of metallic impurities present in the spinach powder, which acts as a sintering aid and leads to increased densification. The BZYMn electrolyte on the other hand shows an opposite trend, with BZYMn synthesized by the green route, resulting in slightly lower densification than the material synthesized by the chemical route due to high concentration of Mn that already exist in spinach resulting in the slightly lower densification (Irshad et al., 2022). The overall densification order for BZYM (M = Co, Fe, Mn) electrolytes synthesized by both green and chemical routes can be represented as BZYMn < BZYFe < BZYCo indicating that Co doping acted as a slightly better sintering aid compared to Fe and Mn because Co doping can help to control the grain growth resulting in a fine-grained microstructure, as can also be seen from the micrographs too (Rehman et al., 2021).

### 3.3 Elemental composition analysis

**FIGURE 4:**
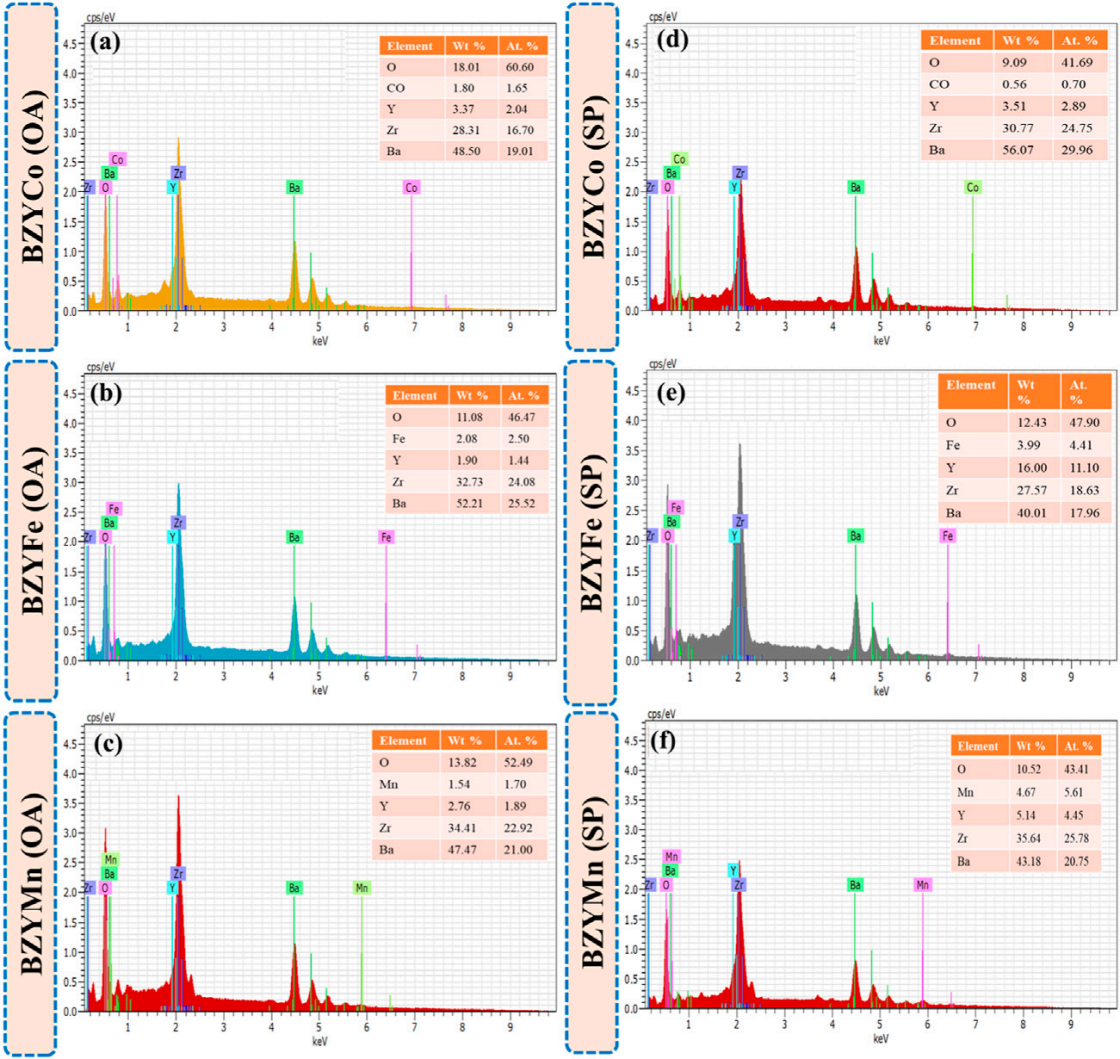
EDS qualitative with quantitative inset analysis of BZYM (M = Co, Fe, Mn) electrolytes using green **(A–C)** and chemical **(E–F)** route.

Figures 4A–F show the EDS spectra of sintered BZYM (M = Co, Fe, Mn) perovskite electrolytes synthesized using spinach and oxalic acid. The compositional analysis of BZYM (M = Co, Fe, Mn) confirms the presence of Ba, Zr, Y, and transition metal dopants (Co, Fe, Mn). The insets in Figures 4A–F show the quantitative analysis of BZYM (M = Co, Fe, Mn) for oxalic acid and spinach powder. It can be observed that no significant impurities were present in BZYM (M = Co, Fe, Mn) synthesized by the green route implying that spinach can be successfully used as chelating agent for the synthesis of BaZr_0.87_Y_0.1_M_0.03_O_3−δ_ electrolyte without any significant impurities.

### 3.4 Spectroscopic analysis

**FIGURE 5:**
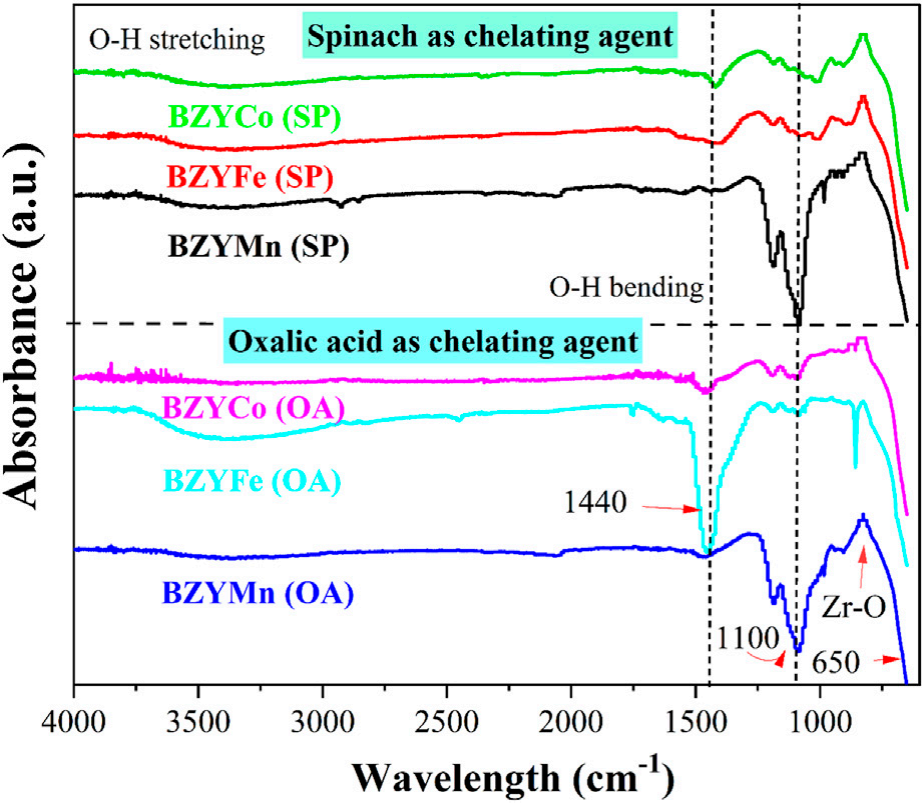
FTIR spectra of BZYM (M = Co, Fe, Mn) electrolytes synthesized by green and chemical route.

Figure 5 presents the FTIR spectra of BZYM (M = Mn, Co, and Fe) perovskite electrolytes synthesized through chemical and green routes. The absorption bands at 3,800–3,200 cm^−1^ and 1700–1,400 cm^−1^ correspond to OH stretching and bending, respectively. These bands indicate the incorporation of water into the lattice which may be due to the atmospheric humidity (moisture). The peaks at 1,500–1,100 cm^−1^ indicate the formation of metal-oxygen-metal bonds such as Ba−O−Ba, Mn−O−Mn, Fe−O−Fe, etc. For Fe-doped BZY, broad and high intensity peaks in the 1,500–1,400 cm^−1^ region are characteristic of Fe−O bond formation (Jincy and Meena, 2022). The intensity of BZYFe electrolyte synthesized with oxalic acid is greater compared to BZYFe electrolyte synthesized with spinach powder, indicating the increased structural disorder (Irshad et al., 2022). The Mn-doped BZY for both chelating agents shows an intense absorption peak at about 1,100 cm^−1^ which is characteristic of Mn−O bond formation. The absorption peak at about 1,200 cm^−1^ indicates C−O stretching. The absorption bands at 400–800 cm^−1^ indicate stretching vibrations of metal-oxygen bonds such as Mn−O, Fe−O, etc. The presence of metal-oxide bond peaks confirms the formation of transition metal doped BZY for both chemical and green synthesis routes.

### 3.5 Thermal analysis

**FIGURE 6:**
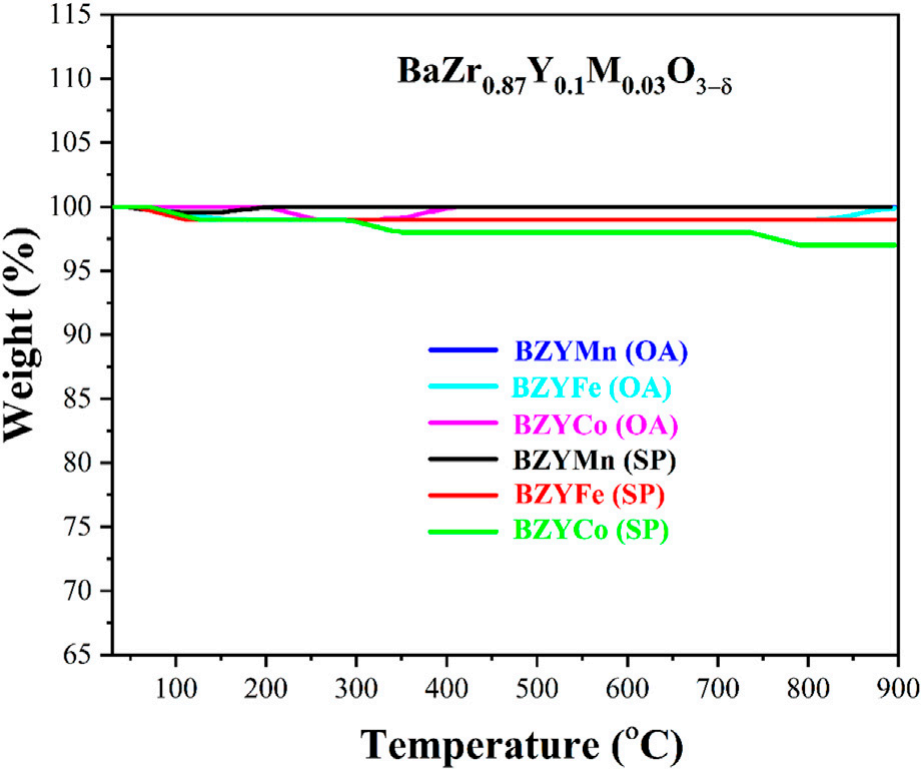
Thermogravimetric curves for BZYM (M = Co, Fe, Mn) electrolytes synthesized by chemical and green route.

Figure 6 shows the thermal analysis of sintered BZYM (M = Co, Fe, Mn) perovskite electrolyte. The TGA graph shows the weight loss in % over the temperature range 30°C–900°C. A minimal amount of weight loss is observed as the TGA was performed after sintering. The process of sintering has already removed the residual water and decomposed the nitrates and other organic impurities. Small weight losses of about 1%–3% may occur due to the incorporation of moisture or impurities into the synthesized materials after sintering. It can be inferred from no prominent weight loss that the BZYM (M = Co, Fe, Mn) electrolytes synthesized through chemical and green routes are thermally stable in the solid oxide fuel cell operating temperature range.

### 3.6 Conductivity

**FIGURE 7:**
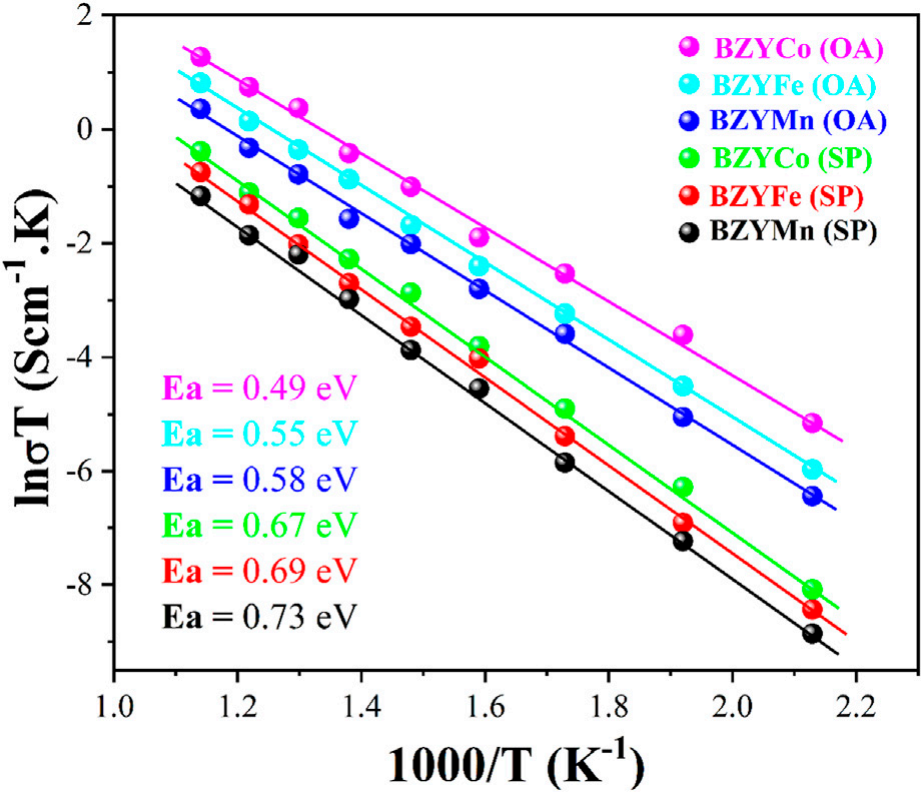
Arrhenius plot of BZYM (M = Co, Fe, Mn) electrolytes synthesized by chemical and green route.

Figure 7 shows the Arrhenius plot for the BZYM (M = Co, Fe, Mn) perovskite electrolytes synthesized with chemical and organic chelating agents. It can be seen from the plot that regardless of the synthesis approach and dopants, all electrolytes exhibited significant conductivities which can be attributed to the fact that the addition of Co, Fe and Mn as the sintering aids lowers the sintering temperature and enhances the p-type conductivity as previously reported (Park et al., 2015).

It is clear from the plot that BZYCo (OA), BZYFe (OA) and BZYMn (OA) electrolytes showed better conductivity compared to electrolytes synthesized with spinach as a chelating agent. The lower conductivity of the BZYCo (SP), BZYFe (SP) and BZYMn (SP) electrolytes can be attributed to metallic impurities present in the spinach and the synergistic effect of both these impurities and dopants (M = Co, Fe, Mn) may have resulted in the lower ionic conductivity of the electrolytes, as these metals reduce the effective concentration of protons, acting as a proton trap, residing on the tetravalent Zr sites. It is well known that the conductivity of the electrolytes in air is attributed to electron-hole conduction and therefore the presence of these metals will result in a lower ionic conductivity (Tao and Irvine, 2007; Park et al., 2015). Nevertheless, the inclusion of sintering aids in the BZY solid solutions elevates the vacancy concentration of oxygen and barium sites, as previously stated (Huang et al., 2010; Benamira et al., 2011). It can also be observed from the plot that BZYCo (OA) and BZYCo (SP) exhibit high conductivity, because Co doping develops oxygen vacancies by replacing Zr at the B-site and these vacancies allow for faster oxygen ion transport, therefore resulting in higher ionic conductivity (Balaguer et al., 2022). The activation energies of the BZYCO (OA), BZYFe (OA) and BZYMn (OA) are 0.49, 0.55, and 0.58 eV respectively, while those of BZYCO (SP), BZYFe (SP) and BZYMn (SP) are 0.67, 0.69, and 0.73 eV respectively. The oxalic acid was used in the pure chemical form without any impurities as a reducing agent, however, spinach uses both oxalic acid (present in higher content) and biomolecules as a reducing and capping agent. Furthermore, spinach also carries metallic impurities that may act as a sintering aid along with a change in electrical properties causing a change in the activation energies compared to chemically synthesized BZYM electrolytes. It indicates that BZYM can be successfully used as an electrolyte despite the presence of impurities in the organic chelate.

### 3.7 Electrochemical performance

**FIGURE 8:**
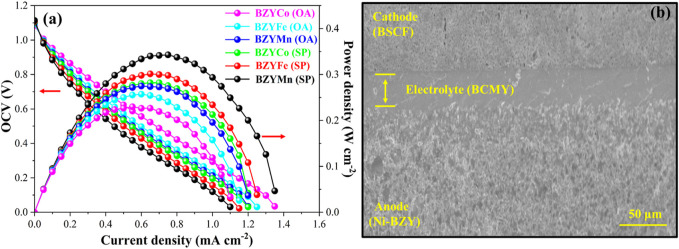
Electrochemical performance of cells at 650°C with BZYM (M = Co, Fe, Mn) electrolytes synthesized by green and chemical route **(A)**; along with the cross-sectional SEM images of anode supported cell (Ni-BZY | BCYM | BSCF) **(B)**.

The electrochemical performance at 650°C for button cells with BZYM (M = Co, Fe, Mn) electrolytes synthesized through chemical and green routes is shown in Figure 8A. The maximum power densities of 344, 293, and 282 mW·cm^−2^ were achieved for the cells with the BZYCO (OA), BZYFe (OA) and BZYMn (OA) respectively. While cells with the BZYCO (SP), BZYFe (SP) and BZYMn (SP) electrolytes, exhibited power densities of 271, 265, and 218 mW·cm^−2^, respectively, indicating that BZYM (M = Co, Fe, Mn) synthesized by both routes can be used as IT-SOFC electrolytes. It can be observed that the obtained power densities are lower than some of the reported values and can be ascribed to the low sintering temperature than the reported sintering temperatures. It is also clear that cells having BZYCo electrolytes synthesized with both routes shows higher power density compared to BZYFe and BZYMn synthesized with corresponding synthesis routes which can be attributed to its dense structure as seen from the surface morphology and the development of oxygen vacancies by replacing the Zr at the B-site, thus resulting in better performance (Gao et al., 2023).

It can be further observed from Figure 8A that the BZYCO (SP), BZYFe (SP) and BZYMn (SP) electrolytes exhibited low power densities compared to the BZYCO (OA), BZYFe (OA) and BZYMn (OA) electrolytes which can be linked to metallic impurities present in spinach causing the formation of structural defects that alter the electrical properties and hence the performance. However, extensive investigation and optimization is still required to fully understand the mechanism that affects the properties of the electrolytes due to metallic impurities present in the organic. Figure 8B shows the cross-sectional SEM images of the anode-supported half-button cell, utilizing BSCF and Ni-BZY as the cathode and anode, respectively. The cross-section reveals a densely structured electrolyte with a well-established connection between the anode and the electrolyte. Overall, it can be concluded that BZYM (M = Co, Fe, Mn) synthesized via sintering aids using this sustainable approach can be successfully used as IT-SOFC electrolytes.

## 4 Conclusion

In summary, for the first time a sustainable approach is successfully employed to synthesize BZYM perovskite electrolytes with sintering aids (Co, Fe, Mn) using spinach without significantly compromising its properties as an electrolyte for SOFC applications. The cubic perovskite structure was confirmed by the XRD with no prominent secondary phases, while the plane shift was due to mismatched ionic radii of dopants, host and impurities. The compositional study confirmed the existence of Ba, Zr and Y together with their respective sintering aids in each sample. Surface micrographs revealed a dense microstructure for all synthesized materials with slight differences in morphology, while the formation of a perovskite structure was also confirmed by the FTIR. The chemically synthesized BZYM (M = Co, Fe, Mn) electrolytes exhibited higher conductivities compared to the green synthesized BZYM (M = Co, Fe, Mn) electrolytes with BaZr_0.87_Y_0.1_M_0.03_O_3−δ_ (M = Co) electrolytes exhibiting the highest conductivity in both cases. The maximum power density of 271 mW·cm^−2^ is attained for the cell having BZYCo electrolyte synthesized by the sustainable approach, suggesting the effective application of this innovative approach in synthesizing perovskite SOFC electrolytes.

## Data Availability

The raw data supporting the conclusion of this article will be made available by the authors, without undue reservation.
